# 27 years of benthic and coral community dynamics on turbid, highly urbanised reefs off Singapore

**DOI:** 10.1038/srep36260

**Published:** 2016-11-08

**Authors:** J. R. Guest, K. Tun, J. Low, A. Vergés, E. M. Marzinelli, A. H. Campbell, A. G. Bauman, D. A. Feary, L. M. Chou, P. D. Steinberg

**Affiliations:** 1 Centre for Marine Bio-Innovation, School of Biological, Earth and Environmental Sciences, University of New South Wales, Sydney, NSW 2052, Australia; 2 SECORE International, 40 Jalan Anjung 5, Horizon Hills, Nusajaya 79100, Johor Malaysia; 3 National Biodiversity Centre, National Parks Board, 1 Cluny Road, Singapore Botanic Gardens, 259569, Singapore; 4 Evolution & Ecology Research Centre, School of Biological, Earth and Environmental Sciences, University of New South Wales, Sydney, NSW 2052, Australia; 5 Sydney Institute of Marine Science, 19 Chowder Bay Rd, Mosman, NSW 2088, Australia; 6 Experimental Marine Ecology Laboratory, Department of Biological Science, National University of Singapore, 117543, Singapore; 7 School of Life Sciences, University of Nottingham, NG7 2UH, United Kingdom; 8 Tropical Marine Science Institute, National University of Singapore, S2S, 18 Kent Ridge Road, 119227, Singapore; 9 Singapore centre for Life Sciences Engineering, Nanyang Technogical University, 60 Nanyang Drive, SBS-01N-27, 637551, Singapore

## Abstract

Coral cover on reefs is declining globally due to coastal development, overfishing and climate change. Reefs isolated from direct human influence can recover from natural acute disturbances, but little is known about long term recovery of reefs experiencing chronic human disturbances. Here we investigate responses to acute bleaching disturbances on turbid reefs off Singapore, at two depths over a period of 27 years. Coral cover declined and there were marked changes in coral and benthic community structure during the first decade of monitoring at both depths. At shallower reef crest sites (3–4 m), benthic community structure recovered towards pre-disturbance states within a decade. In contrast, there was a net decline in coral cover and continuing shifts in community structure at deeper reef slope sites (6–7 m). There was no evidence of phase shifts to macroalgal dominance but coral habitats at deeper sites were replaced by unstable substrata such as fine sediments and rubble. The persistence of coral dominance at chronically disturbed shallow sites is likely due to an abundance of coral taxa which are tolerant to environmental stress. In addition, high turbidity may interact antagonistically with other disturbances to reduce the impact of thermal stress and limit macroalgal growth rates.

Coral reefs have been impacted by multiple human activities for centuries, with overfishing and coastal land-use changes considered the most deleterious practices^1^,^2^. In addition to these localised (although globally distributed) disturbances, climate change has now emerged as an additional driver of unprecedented change on reefs. In particular, periods of anomalously high sea surface temperatures (SSTs), such as those recorded during 1998^3^, are strongly linked to episodes of catastrophic coral mortality following regional coral bleaching events^4^.

One outcome of increasing human disturbances is that many coral reefs have undergone shifts from coral dominance to alternative, less desirable states^5^,^6^,^7^. The most widely studied example of an undesirable phase shift involves a switch from coral dominance to dominance by fleshy macroalgae^8^, although this is only one of several possible alternative states, with shifts towards algal turfs and other animal biota increasingly being documented^9^,^10^,^11^. In some cases, reef building corals may remain dominant following disturbances, but coral taxonomic community structure may shift towards species capable of tolerating new environmental conditions, resulting in novel assemblages^12^,^13^. The most common such change is a shift in dominance from susceptible taxa with branching morphologies, rapid growth rates and high structural complexity to taxa that have foliose or massive morphologies and slower growth rates^12^,^14^,^15^.

Recovery of coral cover to pre-disturbance levels, within a decade following major acute disturbances, has been demonstrated for relatively isolated Indo-Pacific reefs^16^,^17^,^18^. Less is known, however, about the recovery potential of turbid, marginal reefs close to heavily populated urban centers. It is generally accepted that chronic human disturbances compromise the ability of coral reefs to recover from acute disturbances^19^, although some heavily disturbed reefs appear surprisingly resilient^20^.

The effect of chronic disturbances on an ecosystem are not always additive and in some cases, the total impact is less than the sum of the individual disturbances (i.e., antagonistic)^21^. For example, certain chronic disturbances, such as high turbidity, may offer a degree of protection during periods of thermal stress, by reducing light stress^22^. Furthermore, there is no simple relationship between proximity to high human population densities and reef condition^23^. Uncertainty about the effects of multiple human disturbances on the future of tropical reefs is of great significance to society because productive, coral dominated reefs provide valuable ecological goods and services for many coastal communities^24^,^25^,^26^.

Because of the various and complex life histories of the organisms involved (e.g., long lived, modular, colonial) and the high degree of variation in spatial and temporal scales of certain disturbances (e.g., thermal stress, storms, predator outbreaks), understanding the future of coral reefs requires long-term assessments of benthic community structure under a range of conditions^27^. Quantifying changes in coral taxonomic community structure is also critical, because changes in total coral cover alone can be misleading as a measure of ecosystem state^21^. Long-term (i.e., spanning >20 years) studies of reef benthic *and* coral community structure are lacking for certain biogeographic regions and for reefs adjacent to heavily populated urbanised centers, including the biogeographic region known as the ‘coral triangle’^28^,^29^, where most of the world’s reefs and the highest diversity of coral reef species occur. Many of the reefs in this region are close to very high human population densities, and have experienced extreme urbanisation over a relatively short time period.

Singapore’s ecosystems, situated on the edge of the coral triangle, have experienced multiple human disturbances for decades. In the span of just under 200 years, Singapore has undergone a transformation from a sparsely populated, forest-covered island to a highly urbanised city-state. The population has risen from an estimated 150 people in 1819^30^ to >5.4 million at present (www.singstat.gov.sg/statistics/latest_data.html). The majority of the southern coastline and islands, where Singapore’s coral reefs occur, have undergone reclamation and many of the intertidal flats of the fringing and patch reefs have been lost to make way for petrochemical plants, military and recreational areas^30^. Extensive coastal construction, dredge spillage and modified hydrodynamics have resulted in sedimentation rates and levels of total suspended solids exceeding those considered optimal for tropical reefs^31^,^32^,^33^,^34^. Singapore’s reefs are highly turbid, with current average Secchi depths of ~2 m^35^. Underwater visibility, thought to have been about 10 m in the 1960’s, decreased to around 2 m in the late 1980’s and has remained at approximately that level until present^36^. Eutrophication (i.e., phytoplankton cell counts) is thought to have increased at least 30 fold in the last 60 years^37^, although measured nutrient concentrations are relatively low and there is little spatial variation among islands^37^. Although Singapore’s reefs do not experience typhoons or predation by the crown of thorns starfish *Acanthaster planci*, natural disturbances that have contributed to declines in coral cover on many Indo-Pacific reefs^38^,^39^, they were affected by two major thermal coral bleaching events that occurred in 1998 and 2010^40^. While the transformation of Singapore has had clear, significant, negative impacts on both marine and terrestrial biodiversity^41^, diverse coral communities (>250 coral species) still persist on the fringing reefs that surround most of Singapore’s southern islands^42^. At present these coral communities are restricted to ~8 m depth due to very high light attenuation (i.e., <1% of surface PAR at ~9 m depth)^32^ and are composed of stress tolerant taxa typical of heavily sedimented and turbid waters^33^,^43^.

Here we analyse data from surveys of benthic and coral community structure collected over a period of 27 years (1986–2012) on the reef crests and upper slopes of coral reefs to the south of mainland Singapore. Our overall aims were to assess long-term coral reef community responses to acute natural disturbances (e.g., coral bleaching) against a backdrop of chronic disturbance (sedimentation and turbidity). Specifically we ask the following questions: (i) has cover of coral, macroalgae and other benthos changed over the period of study and did trajectories vary between depths? (ii) Was there evidence of a shift in the dominance of certain life history traits among corals (for example, from susceptible fast growing species towards tolerant slow growing species)? (iii) Was there evidence of phase shifts from coral dominance to an alternative state (e.g., macroalgae, turf algae) following bleaching disturbances?

## Methods

### Long-term monitoring of benthic and coral community structure

Surveys of benthic and coral community structure were conducted at a total of 15 sites to the south of the main island of Singapore between 1986 and 2012. Not all sites were surveyed in all years, although at each site exactly the same survey method was used on each occasion. For a full description of sites and the sampling regime see Supplementary Information (Table S1, S2 and Fig. S1). Surveys were carried out at two depths, hereafter known as: “shallow” (the reef crest, approx. 3–4 m depth) and “deep” (upper reef slope, approx. 6–7 m depth) by laying five replicate 20 m transects parallel to the reef crest. Transects were laid haphazardly on each occasion to ensure independence among time points.

SCUBA divers used the line intercept transect method (LIT)^44^ to estimate benthic percentage cover. The LIT method involves a diver estimating the length, to the nearest centimeter, of different benthic categories lying underneath a tape measure laid across the reef. The benthic categories used were: hard coral, macroalgae (i.e., fleshy seaweeds, e.g., *Sargassum* spp.), epilithic algal turfs (i.e., epilithic algal matrix (EAM)^45^), crustose coralline algae (CCA), sponges, soft corals, zoanthids and other living benthic organisms. “Abiotic” benthic categories (i.e., substrate without conspicuous macrofauna) included recently dead coral, rock, sand, unconsolidated rubble and fine sediments (e.g., clay, silt). The genera and growth forms of all hard coral colonies encountered along each transect were also recorded. These survey data were used to quantify temporal changes in: (i) cover of benthic categories (e.g., macroalgae, hard coral etc.), (ii) overall benthic community structure and (iii) taxonomic coral community structure.

### Data analysis

We performed three types of analyses on the benthic data to examine: (i) the effect of time and depth (and their interaction) on cover of eight benthic categories (hard coral, macroalgae, rubble, EAM, fine sediments, CCA, other substrata [e.g., sand, rock] and other biota [e.g., sponges, zoantharians]), (ii) the effect of time and depth on overall coral taxonomic community structure and (iii) changes in multivariate benthic and coral taxonomic community structure before and after bleaching disturbances at four selected shallow sites.

Changes in percentage cover of hard coral, macroalgae and other benthic categories were modelled as a function of time (using a splines smoothing function for years to account for potential correlation among sampling times for each site), depth (“shallow” vs “deep”) and their interaction using Generalised Additive Mixed Models (GAMM) assuming a binomial distribution, with sites as random effects^46^. GAMMs were fitted instead of Generalised Linear Mixed Models (GLMM) because inspection of the residuals in the model validation showed a clear non-linear pattern^46^. Results of the GAMM analysis confirmed that the relationships between time and benthic cover of coral, macroalgae, EAM, rubble, fine sediments, CCA, other substrata and other biota at both depths were all significantly non-linear (Table 1). Analyses were done using the gamm4 package in R^47^.

To visualise changes over time in overall coral taxonomic community structure, non-metric multidimensional scaling (nMDS) ordinations (averaged over all sites) were produced based on Bray-Curtis similarities of square-root transformed data. To determine potential drivers of community patterns, vectors based on Pearson correlations >0.7 were overlaid on nMDS plots to show which taxa correlated most with change over time (taxa contributing <5% to overall cover were removed)^48^.

During the study period, two thermal bleaching events occurred on Singapore’s reefs^40^,^49^. The first documented bleaching event in Singapore was in 1998 during the El Niño event that caused major thermal stress and bleaching related mortality on many reefs in the region^40^,^50^. The second event occurred in 2010 during a thermal anomaly that severely affected reefs throughout the Andaman Sea and parts of the South China Sea^40^,^51^,^52^. The potential of a chronically disturbed system to recover from a bleaching disturbance was analysed using benthic data from four of the shallow sites (H2, HW1, R2 and S2) (Supplementary Information, Table S1, S2, Fig. S1). Only this subset of sites was used because, among the 15 survey sites, they were the only ones surveyed multiple times within each time period, thus allowing us to decouple bleaching effects from natural temporal variability in community structure. Results from the deep sites were not analysed in this way because there was no evidence of recovery post 1998 at these sites (see Results & Supplementary Information Table S2). Overall benthic community structure and coral community structure were compared among 3 time periods using a 2-factor permutational multivariate analyses of variance in the PERMANOVA+ add-on in PRIMER v6 (factors: i] Period, fixed with 3 levels, ii] Years nested in Period)^53^. The time periods were (1) before the first bleaching event (‘pre-1997–98’: years 1988–1993), (2) after the first bleaching event but before the second bleaching event (‘post-1998/pre-2010’: years 1999–2009), and (3) after the second bleaching event (‘post-2010’: years 2011–2012). Bray-Curtis similarity matrices were calculated on square-root transformed data and analyses used 9999 permutations of residuals under a reduced model. SIMPER was used to determine the benthic categories and taxa contributing most to observed differences across periods^53^.

## Results

### Temporal dynamics of cover of hard coral, macroalgae and other benthic categories

**Figure 1:**
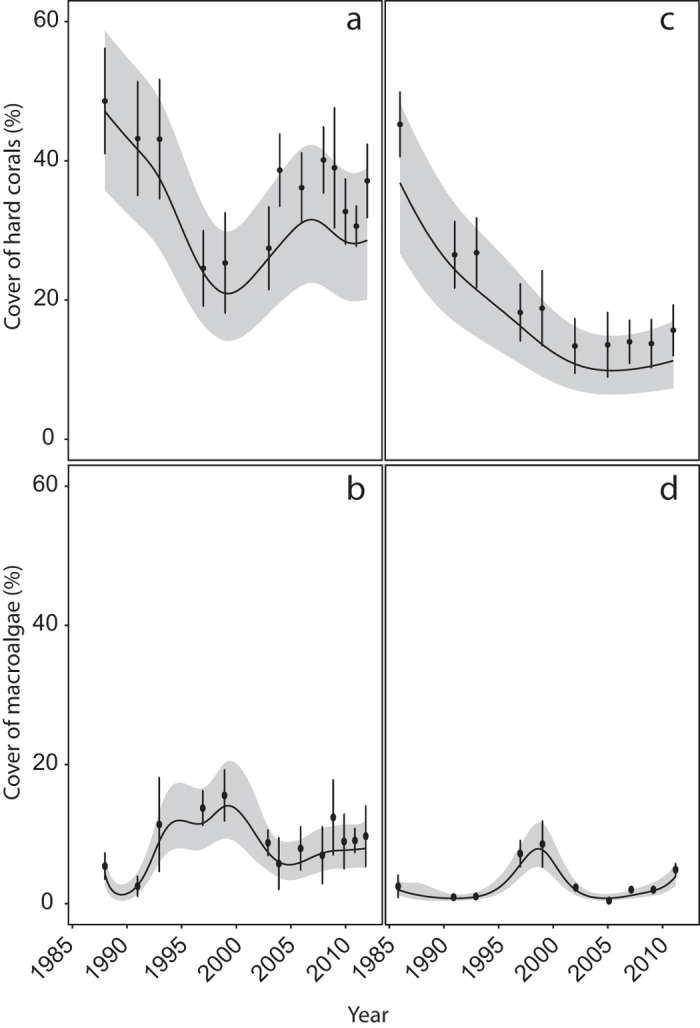
Changes in mean % cover (±SE, n = 6–10) of hard coral (**a**,**c**) and macro algae (**b,d**) at shallow (**a,b**) and deep sites (**c,d**). The fitted line is the predicted covers based on the binomial GAMM model (see Methods), shaded areas are confidence intervals.

**Figure 2:**
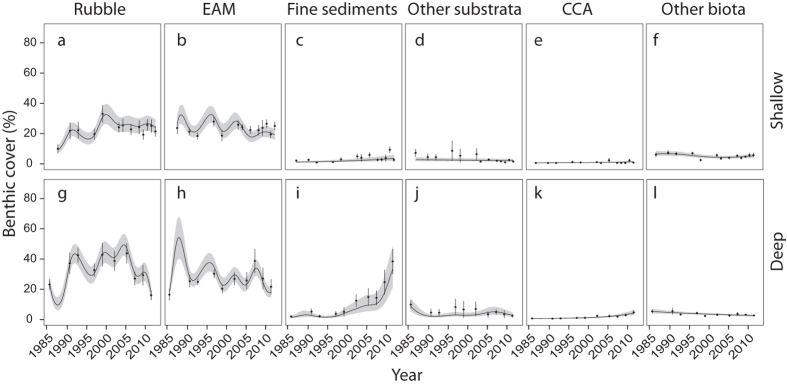
Changes in mean % cover (±SE, n = 6–10) of rubble (**a,g**), EAM (**b,h**), fine sediments (**c**,**i**), other substrata (**d**,**j**), CCA (**e**,**k**) and other biota (**f**,**l**) at shallow (**a–f**) and deep sites (**g–l**). The fitted line is the predicted covers based on the binomial GAMM model (see Methods), shaded areas are confidence intervals.

The relationship between time and benthic cover differed between deep and shallow sites for hard coral, macroalgae, EAM, rubble and fine sediments (significant Year* Depth interaction, Table 1). Between 1988 and 2012 there was an overall ~12% decline in mean coral cover at the shallow sites, whereas at deep sites there was a decline of almost 30% between 1986 and 2012. In contrast, over the same time periods, mean cover of macroalgae only increased by ~4% at the shallow sites and by only ~1% at deep sites (Fig. 1, Supplementary Information, Tables S1 and S2). At shallow sites, mean coral cover among years ranged from ~25% to ~49% with the highest coral cover recorded when monitoring started (1988–1990) and the lowest coral cover recorded in 1997–1998. At shallow sites the greatest decline in coral cover occurred during the decade between 1988 and 1998 (2.41% y^−1^), but by 2008, coral cover had recovered to approximately 1993 levels (~40%) although a second, smaller decline in coral cover of ~9% occurred between 2009 and 2011 (Fig. 1a, Supplementary Information, Table S1). The largest increase in mean macroalgal cover (from ~3% to ~16%) occurred between 1988/1990 and 1999/2000 corresponding with the largest decline in coral cover (Fig. 1b, Supplementary Information, Table S1). Average cover of rubble increased from ~9% in 1988/1990 to ~32% in 1999/2000, but decreased to ~20% by the end of the study (Fig. 2a). At shallow sites, cover of other benthic categories (EAM, fine sediments, other substrata, CCA and other biota) varied from year to year, but did not show an overall decline or increase between the beginning and end of the study (Fig. 2b–f).

At the deep sites, mean coral cover among years ranged from ~13% to ~45% with the highest coral cover recorded during the first time period (1986–1987) and the lowest coral cover recorded in 2003. The greatest decline in mean coral cover occurred between 1986/1987 and 2003 (1.90% y^−1^), after which time coral cover remained relatively stable (ranging from ~13 to 16% total cover), with no recovery to historical levels (Fig. 1c, Supplementary Information, Table S2). Mean cover of macroalgae generally remained very low at deep sites (<4%) with highest cover found between 1997 & 2000 when it increased to ~8% (Fig. 1d, Supplementary Information, Table S2). Mean cover of both rubble and EAM approximately doubled during the first decade of the study, but by the end of the study, cover had declined and returned close to historical levels (Fig. 2g,h). In contrast, mean cover of fine sediments increased markedly from <2% in 1986/1987 to ~25% by the end of the study. Mean cover of other substrata, CCA and other biota varied annually but did not decline or decrease markedly over the course of the study (Fig. 2i–l).

### Temporal changes in coral community structure

On shallow reefs, a total of 53 hard coral genera were recorded during the study period. Averaging across all years, just over half (51%) of the coral community (in terms of relative cover) was comprised of seven genera (*Merulina* 12%, *Pectinia* 11%, *Montipora* 7%, *Pachyseris* 7%, *Porites* 6%, *Echinopora* 4% and *Platygyra* 4%). Average cover of *Acropora* across all years was <1%. The dominant coral growth forms, in terms of cover, were foliose (45%), massive (23%), sub-massive (13%) and encrusting (11%). Branching corals made up <2% of coral cover on average. At deep sites, 54 coral genera were recorded, with 51% of the coral community dominated by five genera (*Pectinia* 19%, *Pachyseris* 14%, *Merulina* 6%, *Porites* 6% and *Montipora* 6%). Average relative cover of *Acropora* across all years was 0.25%. The coral growth forms that dominated the community were foliose (57%), encrusting (20%), massive (11%) and sub-massive (7%). Branching corals made up <1% of coral cover on average.

**Figure 3:**
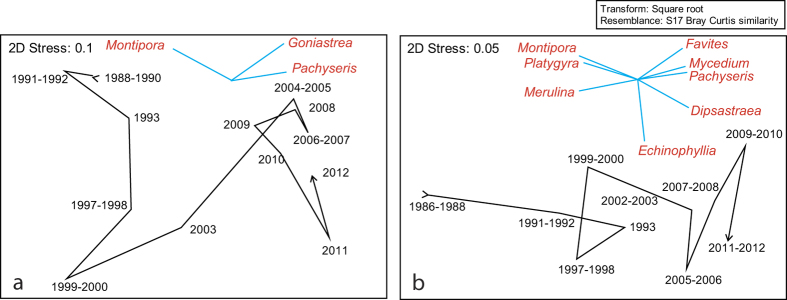
Changes in coral community structure (average of sites) over time visualized as two dimensional non-metric Multidimensional Scaling Plots (nMDS) for shallow (**a**) and deep (**b**) sites. Data are square root transformed and 2D stress is reported. The star diagrams show vectors for cover of coral taxa that correlated most strongly with change (Pearson correlations >0.7).

**Figure 4:**
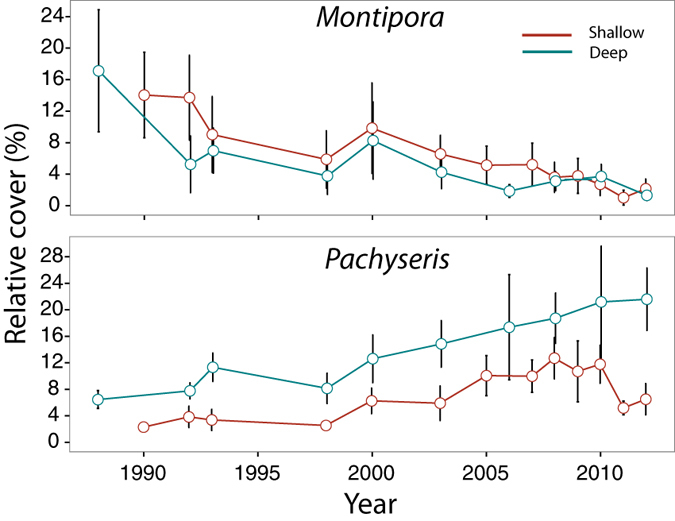
Change in relative cover (%) of the genera *Montipora* (top) and *Pachyseris* (bottom) at shallow (red line) and deep (blue line) sites over the study period. Data are shown from these two genera because they showed the greatest decrease and increase respectively of all coral genera.

Coral community structure, when averaged across all sites, changed over time at both shallow and deep sites, but the pattern of change between shallow and deep sites differed (Fig. 3). At both shallow and deep sites, the genus *Montipora* showed the greatest decline in relative cover over time from ~14–2% and ~17–1% respectively, whereas the genus *Pachyseris* had the greatest relative increase from ~2–12% (in 2010) and ~8–22% respectively (Fig. 4). Relative cover of *Pachyseris* declined from ~12% to 6% at shallow sites following severe bleaching in 2010 (Fig. 4). Coral community structure at shallow sites changed markedly between 1988 and 2000, with a smaller shift occurring in 2011, and these correlated most strongly with reductions in cover of the genus *Montipora* (Fig. 4). Community structure continued to change after 2000 (correlated with increases in *Goniastrea* and *Pachyseris*) (Figs 3 and 4); however there was no return towards pre-1998 structure (Fig. 3), although community structure remained similar between 2004 and 2010 (Fig. 3). At deep sites, change between 1986 and 2012 was driven by reductions in cover of *Montipora*, *Platygyra* and *Merulina* and increases in several genera, including *Favites*, *Mycedium*, *Pachyseris*, *Dipsastraea* (formerly *Favia*) and *Echinophyllia*. There was no evidence of a reverse in the direction of change towards historical structure (Fig. 3).

### Recovery of benthic and coral community structure at shallow sites before and after bleaching

**Figure 5:**
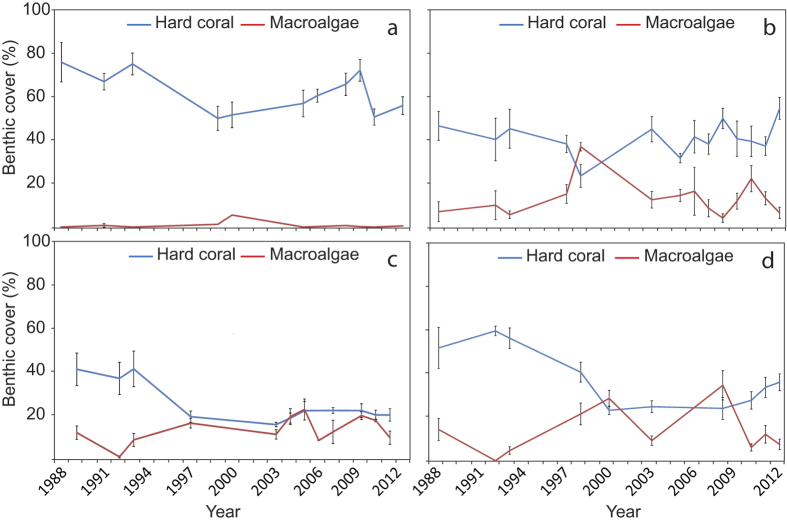
Changes in benthic cover of coral and macroalgae at four sites used in the analysis of community structure pre and post bleaching. Graphs show changes in mean cover of hard coral (blue line) and macroalgae (red line) for shallow sites at (**a**) R2, (**b**) H2, (**c**) HW1, (**d**) S2. Error bars are SE.

At three of the four selected sites (H2, HW1 & S2), benthic community structure differed significantly before and after the first bleaching event (1998; PERMANOVA, H2: pseudo-*F*_2,7_ = 3.4, HW1: pseudo-*F*_2,5_ = 4.4, S2: pseudo-*F*_2,5_ = 3.0, *p* < 0.05 for all three sites) (Supplementary Information, Table S3), but there were no significant differences before the first bleaching event in 1998 and after the second bleaching event in 2010. There were also no significant differences before and after the second bleaching event (PERMANOVA pairwise tests, *p* *>* 0.05) (Supplementary Information, Table S3). Reductions in cover of hard coral and increases in cover of macroalagae and rubble made the greatest contribution to dissimilarity before and after the first bleaching event (SIMPER) (Supplementary Information, Table S4) (Fig. 5).

For coral taxonomic community structure there were significant changes at 2 of the 4 shallow sites (HW1 & R2) before and after bleaching events (PERMANOVA, HW1: pseudo-*F*_2,4_ = 5.1, R2: pseudo-*F*_2,6_ = 3.6, *p* < 0.001 for both sites) (Supplementary Information, Table S5). Coral communities surveyed before the first bleaching event differed from those in-between bleaching events and those after the second bleaching, but there were no significant differences before and after the second bleaching event (PERMANOVA pairwise tests, Table S5). *Montipora* (at R2) and *Plerogyra* (at HW1) made the greatest contribution to dissimilarity (in terms of decreases in relative cover) between time periods (Supplementary Information, Table S6).

## Discussion

Coral cover on Indo-Pacific reefs has declined dramatically in recent decades with some heavily disturbed reefs undergoing phase shifts from coral to algal dominance^5^,^6^,^16^,^54^,^55^. Reefs that have resisted phase shifts and returned to a coral dominated state have, in some instances, undergone major transitions in coral community structure^12^,^56^,^57^. Coral cover in Singapore also declined at both shallow and deep sites during the study period, with the largest declines occurring during the first decade of monitoring. Despite this, coral cover at shallow reef crest sites showed signs of rapid recovery (~16% increase in overall mean coral cover between 1998 and 2008), while coral cover at deep sites declined until 2003 and has still not recovered to historical levels. Despite decades of human disturbance, average coral cover at the shallow reef crest (~36%) is above the current average (~22–27%) for the Indo-Pacific^54^,^58^,^59^, although similar to estimated averages for reefs in the South China Sea region (~40%)^58^. There was clear evidence of a temporal change in coral community structure, but communities were dominated by stress-tolerant and generalist taxa throughout the study period. Susceptible, competitive branching coral taxa were rare even at the onset of the study suggesting that they were never common or were extirpated before long term monitoring began. Furthermore, there was no evidence of phase shifts from coral to macroalgal dominance at any of the sites, despite the fact that adjacent reef flats (~0–2 m depth) have been dominated by erect fleshy macroalgae (e.g., *Sargassum* spp.) since at least the 1970’s^60^. Cover of fine sediments increased markedly at deeper sites and was greater than that of hard corals by 2012, suggesting a shift from a coral-dominated regime to a coral/fine sediment regime at the greater depths.

Much of the global decline on coral reefs has been attributed to coastal land use change, overfishing (leading to reduced herbivory), outbreaks of coral predators and climate change disturbances^1^,^2^. In Singapore we suggest that observed declines in coral cover at shallow and deep sites during the first decade of monitoring were due to a combination of increased sedimentation or turbidity and bleaching induced mortality^30^,^40^,^49^. At the time that monitoring started in the 1980s, Singapore’s reefs were already highly turbid due to almost four decades of extensive coastal development^30^. Between 1988 and 1993, however, approximately 1.5 million m^3^ of clay spoils were dumped on the eastern side of P. Semakau with no attempts made to contain their spread (Supplementary material Appendix 1, Fig. A1). This reportedly led to a thick layer of fine sediments being deposited on nearby reefs^61^. A parsimonious explanation for declines in coral cover prior to 1998 is that this event increased already high levels of sedimentation and turbidity well beyond the healthy range for most corals for a period of a few years^30^,^62^. Several additional major reclamation works have occurred since this event^63^, but they have been accompanied by extensive environmental impact assessments to identify necessary mitigation measures (e.g., use of sediment screens)^64^. To the best of our knowledge, the 1988–1993 event was the only “acute” sediment event during the study period; however, reefs were continually subjected to chronic sedimentation and turbidity throughout.

Further observed declines in coral cover at shallow sites during 1998 and 2010 coincided with two thermal bleaching events and were most likely a result of bleaching induced mortality^40^. Visual estimates in 1998 suggested 25% of coral colonies died due to bleaching (K. Tun, pers. comm) in Singapore. In contrast, surveys conducted during the 2010 bleaching event found ~60% of colonies moderately or severely bleached^40^,^49^, but estimated that only ~4% of colonies died due to bleaching. Other major acute disturbances present on reefs elsewhere, such as crown-of-thorns starfish or cyclonic storms, are absent in Singapore.

The marked contrast in recovery patterns of shallow reef crest sites versus deeper reef slope sites to these combined disturbances is remarkable, particularly when we consider that the reef crest and upper reef slope sites are separated spatially by only a few meters. One of the main drivers of rapid recovery of coral cover on reefs is recruitment of new corals to bare substratum^17^. Measured coral larval settlement rates are relatively low in Singapore^65^, therefore, this process is unlikely to have driven recovery of coral cover at shallow sites. In contrast, many common coral taxa in Singapore (e.g., *Merulina*) tend to suffer partial rather than whole colony mortality and are capable of recovering by rapid horizontal growth^49^,^66^. A plausible explanation therefore, is that the observed recovery at shallow sites following disturbance was driven primarily by regrowth of remnant colonies. Indeed this process is important in other locations following bleaching induced mortality^67^,^68^. Unfortunately we have no data on coral growth or larval settlement rates at deeper sites, making it difficult to determine why deep corals have failed to recover. Poor recovery may in part be due to low light levels as light attenuation is very rapid with <1% of surface PAR reaching the lower reef slope (~9 m depth) resulting in corals being net heterotrophs below 4 m depth^32^,^69^. Furthermore, a high proportion of the benthos is now composed of unstable substrata (e.g., fine sediments, rubble) that would prevent coral larvae from settling or surviving post settlement^69^. The source of the fine sediments that replaced lost coral habitat at deeper sites has not been determined, but may result from low rates of resuspension typical of deeper reef slope habitats^70^.

Deeper reefs in other locations have been proposed as climate change refugia^51^, as corals there may escape bleaching during thermal anomalies due to cooler upwelling water or lower levels of light stress^51^. Clearly this is not the case for deeper reefs in the present study as coral cover declined markedly and was replaced by unstable substrata. Deep water refugia, therefore, may be confined to locations with sufficient light penetration at depth^51^ or for species that can grow at relatively low levels of irradiance (e.g., *Millepora intricata*^71^).

At shallow sites, relatively rapid recovery of coral community structure in the present study may be partly due to the dominance of stress-tolerant (e.g., massive *Porites* and *Platygyra*) or generalist coral taxa (e.g., *Merulina*, *Pachyseris* and *Echinopora*). Stress tolerant taxa are slow growing, have high fecundity, are long lived and relatively less susceptible to thermal stress, whereas competitive and weedy taxa tend to be faster growing, more structurally complex but less tolerant to disturbances^12^. Generalist taxa tend to have traits that overlap both life history groups (i.e., high stress tolerance *and* rapid growth) and these were some of the most dominant taxa in Singapore^12^. Competitive (e.g., *Acropora* and *Turbinaria*) and weedy taxa (e.g., *Pocillopora damicornis*), on the other hand, made up <4% of the overall relative coral cover across years.

Change in coral taxonomic community structure over the course of the study was strongly correlated with reductions in cover of *Montipora* (from ~15% to ~2% relative cover at both shallow and deep sites), a genus that contains competitive, stress tolerant and generalist species^72^. Currently, the most common *Montipora* species on Singapore’s reefs are classified as generalists (e.g., *M. danae* and *M. monasteriata*)^42^,^72^. In the absence of species level identification, we cannot know whether this has always been the case or if competitive species (e.g., *M. digitata*) were more common prior to disturbances.

Most shallow coral communities in this region are dominated by *Acropora*, but this genus was not abundant at any time during the study. Thus there was no evidence of a shift from communities dominated by competitive taxa to dominance by stress-tolerant or generalist taxa, as has been found at other Indo-Pacific sites^21^,^57^. It is probable that tolerant taxa were present prior to the onset of major coastal development in the 1960s, i.e., reefs were “pre-adapted” to tolerate the reductions in water quality^73^. It is also possible that a community shift towards dominance by stress tolerant coral taxa occurred prior to the 1980s and the beginning of these surveys - as has been demonstrated from paleoecological reconstructions of other Indo-Pacific reefs subjected to anthropogenic disturbances decades before the start of ecological monitoring^74^.

There was no evidence of a phase shift from coral to macroalgal dominance at any of the sites. Cover of macroalgae was low throughout the study period and increased only marginally. EAM also remained relatively stable at both shallow and deep sites over the 27 years of study on Singapore’s reefs. The average biomass of herbivorous fish in Singapore is estimated to be seven times lower (~4 g m^−2^) than the average for the broader Indo-Pacific (~29 g m^−2^)^59^,^75^. Densities of urchins are also low, although within the range found for Indo-Pacific reefs, but there is no relationship between macroalgal cover and urchin densities^59^,^75^. Combined, these data suggest that processes other than herbivory are responsible for maintenance of low macroalgal cover on the study reefs^59^. Our findings also support the contention that the EAM constitutes a stable regime on turbid reefs, possibly as a result of herbivory suppression under sediment-laden conditions^77^.

The presence of chronic human disturbances, the relatively high coral cover and rapid recovery following bleaching in 1998 suggest that disturbances may act antagonistically on Singapore’s shallow reefs. For example, light attenuation in Singapore’s turbid coastal waters results in an almost 50% reduction in photosynthetic efficiency of *Sargassum* at the reef crest^78^. If algal growth is limited by light even at quite shallow depths then relatively low rates of herbivory^59^ may be sufficient to prevent macroalgae from overgrowing coral dominated areas. Similarly, while adaptation and acclimatisation following more severe thermal bleaching events (e.g. in 1998) have already been suggested as parsimonious explanations for the present level of thermal tolerance^40^, high levels of turbidity may also promote resistance to bleaching impacts by protecting colonies from stressful levels of irradiance during periods of thermal stress^4^,^22^. If so, these could provide examples of “ecological surprises”, where negative disturbances (e.g., increased turbidity, thermal stress) interact antagonistically, resulting in a lower net impact^79^.

Our data support the notion that coral reefs will change rather than disappear entirely due to coastal land use changes^80^ and provide a glimmer of hope that some heavily disturbed Indo-Pacific reefs can remain in a coral dominated state. Highly urbanised coral assemblages may be restricted to shallower areas and, if present, be dominated by stress-tolerant and generalist coral forms. Degradation does not always occur gradually and phase shifts can occur when a system has reached a tipping point^25^, therefore, the fact that the shallow reef communities in the present study have retained relatively high coral cover for almost three decades does not mean that they will remain this way indefinitely. Furthermore, despite high coral cover, we do not know if these altered shallow coral communities are providing any of the functions and ecological services normally associated with reef ecosystems (e.g., reef building, productive fisheries, diving tourism)^21^. If this is the case then future monitoring programs should consider incorporating other metrics such as net accretion, structural complexity and herbivory rates to assess reef health more broadly^81^.

## Additional Information

**How to cite this article**: Guest, J. R. *et al*. 27 years of benthic and coral community dynamics on turbid, highly urbanised reefs off Singapore. *Sci. Rep*. **6**, 36260; doi: 10.1038/srep36260 (2016).

**Publisher’s note:** Springer Nature remains neutral with regard to jurisdictional claims in published maps and institutional affiliations.

## Supplementary Material

Supplementary Information

## Figures and Tables

**Table 1:** Results of likelihood ratio tests for relationship of time (years) and depths (shallow and deep) with proportion cover of eight measured benthic categories.

Variable	Factor	Chi-square	df	p
Hard coral	Year*Depth	56.00	2.00	***
Macroalgae	Year*Depth	43.40	2.00	***
EAM	Year*Depth	17.10	2.00	***
Rubble	Year*Depth	118.50	2.00	***
Fine sediments	Year*Depth	48.40	2.00	***
Other biota	Year*Depth	0.60	2.00	0.71
	Year	30.40	2.96	***
	Depth	36.90	1.00	***
CCA	Year*Depth	2.30	2.00	0.31
	Year	42.10	2.68	***
	Depth	52.00	1.00	***
Other substrata	Year*Depth	0.00	2.00	1.00
	Year	124.10	7.07	***
	Depth	14.9	1	***

Other substrata = sand, rock and newly dead coral; other benthic = all fauna except hard corals, e.g., sponges, zoanthids etc. ***=P < 0.001.
