# Supplementary material for: 27 years of benthic and coral community dynamics on turbid, highly urbanised reefs off Singapore
Source: Sci Rep. 2016 Nov 8;6:36260. doi: 10.1038/srep36260 (PMC5099948; doi:10.1038/srep36260)
Supplement: Supplementary Information [file srep36260-s1.pdf]

27 years of benthic and coral community dynamics on turbid, highly urbanised reefs off Singapore

JR Guest, K Tun, J Low, A Vergés, EM Marzinelli, AH Campbell, AG Bauman, DA Feary, LM Chou, PD Steinberg

Supporting Information

Methods

Long-term monitoring of benthic and coral community structure

Initially there were a total of 10 sites used for the benthic surveys, however, 6 of these were not resurveyed after 2003 (H1, HW2, L2, L4, R1, S1) and they were replaced by 5 sites (J1, K1, PSD1, PSL3, S4) that were considered similar in terms of coral community structure. The reasons why sites were not resurveyed were purely logistical and included: limited access due to land reclamation works (e.g., major reclamation at sites S1, L2, L4 and H1 prevented access to those sites) and safety concerns due to boat traffic (e.g., site R1). Not all sites were surveyed in all years (see Table S1) resulting in certain years having surveys at only a limited number of sites. Therefore, to ensure that a representative range of sites were included at each time point, sites were pooled within year groups where necessary so that each year group contained at least six sites. Years that were grouped for shallow sites were 1988-1990, 1991-1992, 1997-1998, 1999-2000 & 2006-2007; and for deep sites were 1986-1988, 1991-1992, 1997-1998, 1999-2000, 2002-2003, 2005-2006, 2007-2008, 2009-2010 & 2011-2012.

Results

Average values for benthic categories at shallow and deep sites

On shallow (~3-4 m) reefs the mean cover of hard coral and macroalgae across all years and sites was 35.8% (SD ± 7.2%) and 10.1% (SD ± 3.6%) respectively. The mean cover of EAM was relatively high at 21.8% (SD ± 2.9%), while mean cover of CCA was 0.4% (SD ± 0.60%) and other biota (i.e., sponges, soft corals and zooanthids) was 4.6% (SD ± 1.4%) were relatively low at shallow sites. A high proportion of the benthos (mean cover 21.5 SD ± 5.0%) was composed of unconsolidated dead coral fragments, i.e., rubble, with only 4.6% (SD ±1.4%) composed of other abiotic substrata (e.g., sand, silt, rock). On deep (~6-7 m) reefs, mean coral cover across all years was 20.8% (SD ± 9.8%) and mean cover of macroalgae was 3.1% (SD ± 2.79%). Mean cover of EAM was 23.9% (SD ± 5.1%), cover of CCA was 1.2% (SD ± 1.2%) while mean cover of other biota was 2.9% (SD ± 1.1%). Mean cover of rubble was 31.8% (SD ± 9.1%) while mean cover of other abiotic substrata was 16.6% (SD ± 10.1%) (Table S1 & S2).

Table S1. Percentage cover of hard coral and macroalgae at all shallow sites showing sites and years surveyed. Values are total % cover for each site and year or year group. Numbers in parentheses are SD.

**Hard Coral**														
**Island**	**Site**	**1988-1990**	**1991-1992**	**1993**	**1997-1998**	**1999-2000**	**2003**	**2004-2005**	**2006-2007**	**2008**	**2009**	**2010**	**2011**	**2012**
**Pulau Hantu**	H1	31.3(19.7)	8.4(5.8)	10.2(12.6)	10.2(9.9)		8.6(2.4)							
	H2	49.1(14.0)	48.7(26.5)	41.1(19.6)	36.0(17.4)		46.8(11.8)	34.3(6.8)	39.0(11.5)	46.1(12.5)	39.2(16.0)	38.5(13.7)	36.7(8.4)	48.8(17.8)
**Hantu West**	HW1	41.5(16.6)	37.3(16.5)	41.6(18.2)	19.8(5.8)		16.7(2.3)	20.8(7.4)	22.6(2.3)		22.5(7.0)	20.6(4.9)	20.6(6.4)	
	HW2	70.4(18.9)	63.9(10.3)	61.6(19.7)	33.8(7.8)		43.5(13.9)							
**Pulau Jong**	J1								34.9(7.4)	30.5(5.5)	16.4(12.1)		23.1(8.2)	21.6(6.0)
**Kusu Island**	K1						22.9(8.6)	48.4(17.3)	40.6(12.5)	48.4(10.8)	54.9(10.4)	45.6(18.4)	44.1(11.9)	56.6(12.7)
**Lazarus**	L2	60.8(13.7)	47.3(15.7)	21.6(13.2)	21.6(3.6)	25.9(9.7)								
	L4	2.6(5.4)	3.3(3.0)	7.1(11.3)	0.2(0.4)	1.6(1.5)	4.1(2.7)							
**Sisters Island**	PSD1							39.6(6.4)		41.9(15.1)			29.6(16.7)	29.6(14.3)
	PSL3							32.0(5.9)	27.0(6.9)	34.7(13.2)		25.1(4.1)	21.8(7.1)	26.4(7.1)
**Raffles Lighthouse**	R1	76.5(12.0)	78.3(10.1)	73.4(8.6)	48.4(11.4)	38.1(8.0)	56.2(7.8)							
	R2	75.9(20.2)	67.0(8.6)	75.2(11.2)		50.9(12.1)		56.9(13.7)	60.5(6.7)	65.8(11.5)	72.2(11.1)	50.7(8.4)		56.0(9.1)
**Semakau**	S1	26.1(14.1)	19.1(9.4)	8.9(10.3)	9.9(8.6)	12.2(5.6)	23.4(10.0)							
	S2	51.8(21.1)	59.6(4.8)	56.3(10.5)	40.6(10.3)	23.1(4.2)	24.8(6.1)			24.0(11.2)		27.7(9.0)	33.7(10.3)	36.0(8.7)
	S4								25.9(6.5)	28.8(11.5)	29.5(8.7)	20.3(12.0)	33.8(19.1)	21.3(10.3)
**Macroalgae**														
**Pulau Hantu**	H1	13.3(14.7)	3.3(4.9)	17.4(11.2)	17.2(8.5)		11.4(10.2)							
	H2	7.4(10.1)	10.3(14.8)	6.0(3.8)	26.2(13.4)		12.8(8.4)	14.8(6.2)	12.8(17.8)	4.5(4.5)	12.2(8.1)	22.4(13.3)	13.5(6.7)	6.8(5.7)
**Hantu West**	HW1	12.5(6.9)	1.4(1.1)	9.1(6.6)	16.8(4.9)		13.0(4.4)	21.4(9.1)	12.8(9.2)		20.3(3.9)	18.0(1.4)	10.2(6.8)	
	HW2	0.2(0.4)	0.1(0.3)	0.4(0.8)	14.3(7.6)		4.4(4.0)							
**Pulau Jong**	J1								23.2(18.2)	9.8(7.3)	13.2(2.3)		10.1(6.6)	34.6(12.3)

**Kusu Island**	K1						12.8(7.7)	0.3(0.5)	0.2(0.4)	0.0(0.0)	0.4(0.3)	0.8(1.0)	1.9(1.6)	1.3(0.9)
**Lazarus**	L2	0.0(0.0)	0.5(0.6)	11.5(9.9)	3.2(2.6)	9.9(5.8)								
	L4	9.8(6.8)	11.5(12.9)	2.2(2.0)	9.0(6.5)	20.2(3.1)	19.0(3.4)							
**Sisters Island**	PSD1							2.4(4.0)		1.8(1.9)			12.5(7.4)	0.6(0.5)
	PSL3							0.8(0.8)	3.5(2.7)	2.4(2.8)		0.3(0.3)	2.4(3.8)	9.9(6.1)
**Raffles Lighthouse**	R1	0.0(0.0)	0.7(0.9)	0.0(0.0)	0.9(0.9)	17.2(10.4)	2.4(1.8)							
	R2	0.0(0.0)	0.7(1.5)	0.0(0.0)		3.4(5.5)		0.0(0.0)	0.2(0.3)	0.7(0.8)	0.2(0.3)	0.0(0.0)		0.5(0.4)
**Semakau**	S1	5.7(6.7)	4.9(7.8)	71.1(20.0)	10.5(5.0)	19.5(6.0)	3.6(3.0)							
	S2	14.3(11.5)	0.0(0.0)	4.6(3.9)	21.5(11.2)	28.6(8.3)	9.2(4.9)			34.6(15.0)		6.1(3.7)	12.1(9.0)	7.5(5.4)
	S4								8.7(8.1)	9.0(5.1)	35.0(9.3)	21.8(13.2)	11.6(1.2)	23.5(6.8)

Table S2. Percentage cover of hard coral and macroalgae at all deep sites showing sites and years surveyed. Values are total % cover for all transects for each site and year or year group. Numbers in parentheses are SD.

**Hard Coral**											
**Island**	**Site**	**1986-1987**	**1991-1992**	**1993**	**1997-1998**	**1999-2000**	**2003**	**2005-2006**	**2007-2008**	**2009-2010**	**2011-2012**
**Pulau Hantu**	H1	27.7(17.3)	5.5(9.2)	16.0(16.1)	12.4(5.0)		5.5(5.1)				
	H2	52.1(22.9)	30.0(13.9)	32.9(18.6)	24.2(7.5)		26.2(6.0)	12.4(6.5)	15.5(9.0)	9.0(5.9)	14.0(4.6)
**Hantu West**	HW1	64.1(11.7)	28.0(8.7)	25.0(12.7)	18.9(7.2)		1.6(0.8)	16.1(5.7)	9.4(5.6)	8.8(3.7)	6.3(2.6)
	HW2	37.2(24.0)	48.4(5.7)	45.3(19.6)	27.5(11.1)		4.5(3.2)				
**Pulau Jong**	J1							8.7(4.2)	10.7(7.1)	9.0(5.7)	10.6(7.8)
**Kusu Island**	K1						9.4(3.1)	1.2(1.1)	12.5(11.9)	5.5(3.8)	10.9(7.6)
**Lazarus**	L2		25.7(9.6)	16.5(12.5)	7.0(2.1)	13.2(2.2)					
	L4		2.0(2.2)	0.6(0.4)	1.7(2.5)	2.0(2.9)	1.5(1.9)				
**Sisters Island**	PSD1							2.7(0.8)	6.0(2.1)		7.8(5.6)
	PSL3								7.4(2.8)	10.4(6.4)	9.7(7.4)
**Raffles Lighthouse**	R1	30.6(12.3)	41.6(15.8)	25.8(13.0)	40.5(9.9)	27.4(7.8)	40.6(13.8)				

	R2	45.5(6.5)	40.2(14.2)	55.8(13.3)		40.5(9.6)	18.9(10.6)	38.2(11.4)	37.0(10.5)	33.8(12.7)	41.1(9.9)
**Semakau**	S1	43.8(15.6)	15.3(7.1)	16.9(11.0)	7.9(3.4)	13.3(3.0)	14.3(6.2)				
	S2	61.0(6.3)	30.7(14.5)	32.6(12.7)	22.7(5.7)	17.8(3.5)	11.4(5.4)		15.4(9.8)	22.8(3.3)	25.2(11.0)
	S4							15.3(9.7)	11.7(8.2)	10.8(6.8)	14.6(9,.2)
**Macroalgae**											
**Pulau Hantu**	H1	0.2(0.4)	0.3(0.6)	3.0(3.5)	12.1(9.2)		1.3(1.9)				
	H2	10.9(9.2)	0.3(0.6)	0.1(0.3)	11.6(9.3)		4.0(3.4)	0.2(0.4)	2.8(4.1)	1.3(1.6)	4.2(6.4)
**Hantu West**	HW1	0.0(0.0)	1.0(1.4)	0.6(1.4)	0.0(0.0)		0.0(0.0)	0.3(0.5)	1.5(1.2)	2.1(1.1)	1.9(2.1)
	HW2	0.1(0.2)	0.0(0.0)	0.0(0.0)	14.2(4.6)		0.0(0.0)				
**Pulau Jong**	J1							0.6(0.8)	3.1(4.7)	4.1(0.8)	4.0(4.9)
**Kusu Island**	K1						3.5(1.7)	0.0(0.0)	0.3(0.8)	0.7(1.2)	0.1(0.4)
**Lazarus**	L2		0.4(0.8)	0.0(0.0)	0.5(0.6)	1.7(1.9)					
	L4		0.6(0.5)	2.1(2.3)	11.1(5.1)	9.1(8.2)	4.3(4.5)				
**Sisters Island**	PSD1							0.1(0.2)	0.2(0.5)		8.8(3.9)
	PSL3								1.6(2.6)	4.0(4.2)	8.3(10.6)
**Raffles Lighthouse**	R1	0.0(0.0)	1.8(2.4)	0.0(0.0)	5.8(3.4)	24.3(17.0)	2.3(0.7)				
	R2	0.1(0.3)	0.1(0.3)	0.6(0.9)		7.6(6.1)	3.5(3.1)	0.4(0.2)	0.5(0.8)	1.5(1.1)	2.6(2.4)
**Semakau**	S1	7.4(7.5)	3.0(3.5)	2.3(2.6)	1.5(1.5)	5.6(2.2)	3.8(6.1)				
	S2	0.0(0.0)	0.0(0.0)	0.0(0.0)	2.6(2.0)	2.4(1.7)	0.7(0.9)		0.9(1.3)	0.5(0.6)	1.8(2.2)
	S4							0.0(0.0)	3.5(5.5)	0.0(0.0)	3.8(4.1)

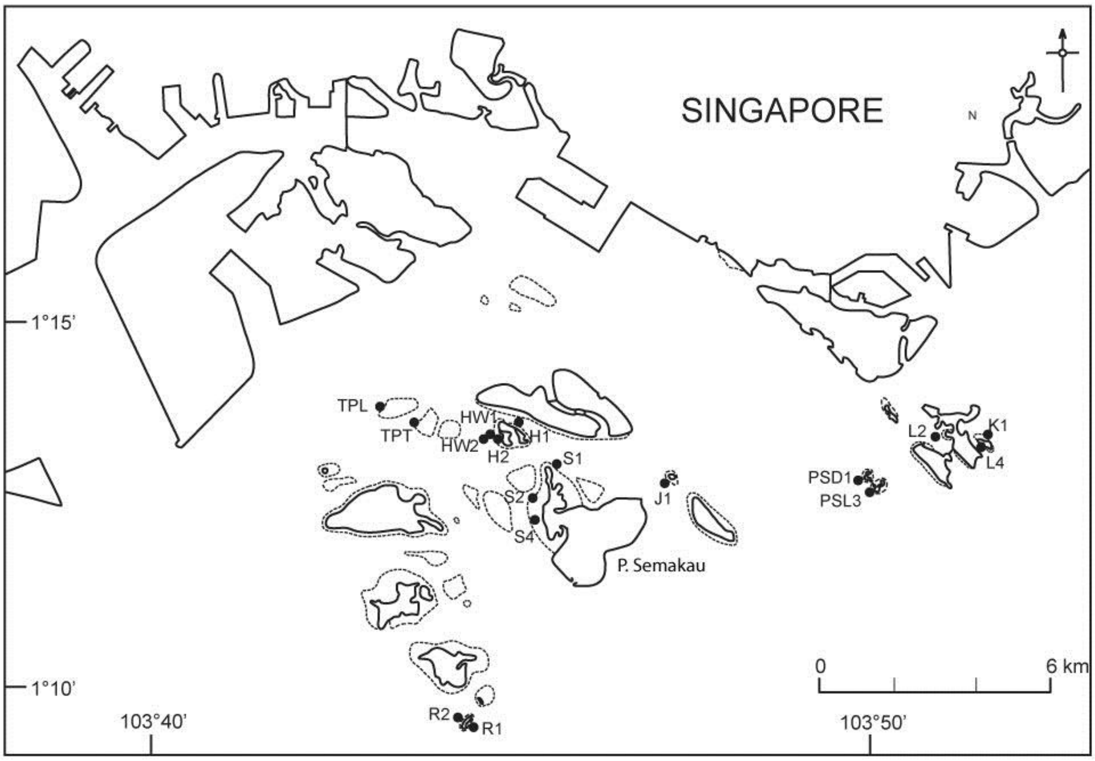

Fig. S1. Map of Singapore's southern islands showing study sites. See Table S1 & S2 for full site names and years when sites were surveyed. The map image is modified from Fig. 1 in Bauman et al. (2015)[59] and used with permission of the author.

Table S3. PERMANOVAs based on Bray-Curtis similarity measure of square-root transformed %covers of benthic communities before, in-between and after bleaching events during the study.*

**Site**						
**H2**	Source	df	SS	MS	Pseudo-F	P(perm)
	Pe	2	3737	1868	3.41	**0.024**
	Ye(Pe)	7	3897	557	1.33	0.129
	Res	45	18802	418		
	Total	54	26431			
	Pairwise tests: 1≠2, 1=3, 2=3					
**HW1**	Source	df	SS	MS	Pseudo-F	P(perm)
	Pe	2	5143	2571	4.36	**0.004**
	Ye(Pe)	5	3005	601	2.58	**0.000**
	Res	34	7930	233		
	Total	41	16484			
	Pairwise tests: 1≠2, 1=3, 2=3					
**R2**	Source	df	SS	MS	Pseudo-F	P(perm)
	Pe	2	2616	1308	2.46	0.078
	Ye(Pe)	6	3300	550	2.62	**0.000**
	Res	39	8176	210		
	Total	47	14478			
**S2**	Source	df	SS	MS	Pseudo-F	P(MC)
	Pe	2	6082	3041	3.01	**0.026**
	Ye(Pe)	5	5056	1011	4.43	**0.000**
	Res	32	7296	228		
	Total	39	18433			
	Pairwise tests: 1≠2, 1=3, 2=3					

*Period (Pe) was fixed with 3 levels ([1] pre-1997-98, [2] post-1998/pre-2010, [3] post-2010), Year (Ye) was random nested in Pe. The replicates were the transects (n=5). P-values were calculated using 9,999 permutations under a reduced model (H2,HW1,R2) or Monte-Carlo (S2).

Table S4. Results of SIMPER for benthic community structure for sites and time periods that had significant PERMANOVA result. Groups are years before and after coral bleaching events: group 1 = pre 1998 bleaching; group 2 = post 1998 bleaching/pre 2010 bleaching.

**Site H2**						
**Average dissimilarity = 32.98**						
	Group 1	Group 2				
**Benthic category**	Av.Abund	Av.Abund	Av.Diss	Diss/SD	Contrib%	Cum.%
**Macroalgae**	2.05	3.01	5.09	1.35	15.43	15.43
**Rubble**	3.15	4.01	4.93	1.31	14.96	30.39
**Sand**	2.45	0.56	4.83	1.53	14.64	45.03
**Hard coral**	6.64	6.31	3.36	1.42	10.18	55.21
**Silt**	0.16	1.52	3.36	1.37	10.17	65.38
**EAM**	3.86	4.32	2.95	1.06	8.96	74.34
**Sponge**	1.25	1.1	2.62	1.47	7.93	82.27
**Other biota**	1.03	0.89	2.37	1.23	7.18	89.46
**Zooanthid**	0.47	0.72	1.86	1.05	5.63	95.09
**Site HW1**						
**Average dissimilarity = 30.38**						
	Group 1	Group 2				
**Benthic category**	Av.Abund	Av.Abund	Av.Diss	Diss/SD	Contrib%	Cum.%
**Rubble**	2.77	5.11	6.08	1.51	20.02	20.02
**Silt**	1.59	2.94	4.43	1.43	14.59	34.61
**Macroalgae**	2.42	4.2	4.43	1.46	14.57	49.18
**Hard coral**	6.19	4.5	4.33	1.65	14.26	63.44
**EAM**	5.42	3.98	3.45	1.45	11.36	74.8
**Sand**	0.55	1.42	2.96	1.2	9.75	84.56
**Sponge**	1.95	1.03	2.83	1.51	9.31	93.87
**Site S2**						
**Average dissimilarity = 36.25**						
	Group 1	Group 2				
**Benthic category**	Av.Abund	Av.Abund	Av.Diss	Diss/SD	Contrib%	Cum.%
**Rubble**	2.41	5.6	7.64	1.69	21.07	21.07
**Macroalgae**	1.81	4.68	7.46	1.6	20.58	41.65
**Hard coral**	7.42	4.84	6.14	2.16	16.94	58.59
**EAM**	4.5	3.39	3.36	1.58	9.26	67.85
**Other biota**	1.51	1.01	2.59	1.47	7.15	75
**Silt**	0.94	0.69	2.5	1.11	6.89	81.89
**Sponge**	1.29	0.78	2.23	1.38	6.14	88.04
**Sand**	0.26	0.66	1.8	0.73	4.96	93

Table S5. PERMANOVAs based on Bray-Curtis similarity measure of square-root transformed %covers of coral taxa before, in-between and after bleaching events during the study.*

**H2**	**Source**	**df**	**SS**	**MS**	**Pseudo-F**	**P(perm)**
	Pe	2	4280.2	2140.1	1.396	0.0802
	Ye(Pe)	7	10740	1534.3	1.0223	0.4214
	Res	36	54029	1500.8		
	Total	45	69061			
**HW1**	Source	df	SS	MS	Pseudo-F	P(MC)
	Pe	2	13941	6970.7	5.0482	**0.0003**
	Ye(Pe)	4	5523.2	1380.8	1.1826	0.2411
	Res	28	32692	1167.6		
	Total	34	52156			
	Pairwise tests: 1≠2=3					
**R2**	Source	df	SS	MS	Pseudo-F	P(perm)
	Pe	2	8154.1	4077.1	3.5699	**0.0001**
	Ye(Pe)	6	6694.5	1115.7	1.0789	0.3102
	Res	34	35161	1034.2		
	Total	42	49930			
	Pairwise tests: 1≠2=3					
**S2**	Source	df	SS	MS	Pseudo-F	P(MC)
	Pe	2	10455	5227.4	1.694	0.071
	Ye(Pe)	5	15429	3085.9	2.7543	**0.0001**
	Res	32	35852	1120.4		
	Total	39	61736			

*Period (Pe) was fixed with 3 levels ([1] pre-1997-98, [2] post-1998/pre-2010, [3] post-2010), Year (Ye) was random nested in Pe. The replicates were the transects (n=5). P-values were calculated using 9,999 permutations under a reduced model (H2,R2) or Monte-Carlo (HW1,S2).

Table S6. Results of SIMPER for taxonomic community structure for sites that had significant PERMANOVA result. Groups are years before and after coral bleaching events: group 1 pre 1998 bleaching; group 2 post 1998 bleaching/pre 2010 bleaching and; group 3 post 2010 bleaching.

**Site HW1**						
**Time periods 1 & 2**						
**Average dissimilarity = 58.86**						
	**Time 1**	**Time 2**				
**Genus**	Av.Abund	Av.Abund	Av.Diss	Diss/SD	Contrib%	Cum.%
Plerogyra	3.77	0.41	5.69	1.62	9.66	9.66
Astreopora	0.24	2.97	4.61	1.56	7.83	17.49
Pavona	2.85	0.66	4.01	1.46	6.8	24.3
Goniastrea	1.67	2.4	2.95	1.32	5.01	29.31
Cyphastrea	1.4	2.76	2.88	1.42	4.9	34.21
Dipsastraea	1.42	2.84	2.85	1.31	4.85	39.06
Fungia	2.03	0.55	2.65	1.51	4.5	43.55
Podabacia	0.47	1.48	2.45	0.95	4.16	47.71
Diploastrea	0.56	1.24	2.41	0.69	4.09	51.8
Pachyseris	0.74	1.73	2.4	1.37	4.07	55.87
Merulina	3.63	3.11	2.39	1.3	4.06	59.94
Echinopora	1.34	0.61	2.21	1.02	3.75	63.69
Platygyra	1	1.34	2.2	1.06	3.74	67.43
Oxypora	1.33	0	2.15	0.92	3.64	71.07
Montipora	0.94	0.72	2.07	0.81	3.53	74.6
Pectinia	3.19	3.49	1.88	1.11	3.2	77.79
Favites	0.41	1.12	1.88	1.05	3.19	80.98
Porites	0.77	1.04	1.74	1.13	2.96	83.94
Hydnophora	0.77	0	1.18	0.79	2	85.94
Galaxea	0.59	0.25	1.07	0.81	1.81	87.75
Goniopora	0.54	0.08	0.91	0.61	1.54	89.29
Leptoseris	0.12	0.37	0.7	0.47	1.19	90.48
**Time periods 1 & 3**						
**Average dissimilarity = 63.08**						
	Time 1	Time 3				
**Genus**	Av.Abund	Av.Abund	Av.Diss	Diss/SD	Contrib%	Cum.%
Plerogyra	3.77	0	6.38	1.75	10.12	10.12
Astreopora	0.24	3.29	5.24	1.49	8.31	18.43
Pavona	2.85	0.74	4.32	1.43	6.85	25.28
Cyphastrea	1.4	2.54	3.86	1.47	6.12	31.4
Favites	0.41	2.33	3.61	1.49	5.72	37.12
Pachyseris	0.74	1.97	3.04	1.28	4.81	41.94
Fungia	2.03	0.28	2.99	1.91	4.74	46.68
Pectinia	3.19	3.67	2.83	1.61	4.48	51.16
Porites	0.77	1.87	2.8	1.31	4.45	55.61

Goniastrea	1.67	0.77	2.54	1.22	4.03	59.64
Echinopora	1.34	1.2	2.4	1.2	3.8	63.44
Dipsastraea	1.42	0.99	2.26	1.34	3.58	67.02
Oxypora	1.33	0	2.24	0.92	3.54	70.57
Diploastrea	0.56	0.97	2.16	0.62	3.43	74
Platygyra	1	0.8	2.05	0.89	3.25	77.24
Merulina	3.63	3.72	1.97	1.08	3.13	80.37
Galaxea	0.59	1.24	1.82	1.5	2.88	83.25
Montipora	0.94	0	1.55	0.65	2.46	85.71
Hydnophora	0.77	0	1.23	0.79	1.95	87.66
Goniopora	0.54	0.3	1.11	0.72	1.76	89.42
Mycedium	0.08	0.53	0.97	0.55	1.54	90.96
**Site R2**						
**Time periods 1 & 2**						
**Average dissimilarity = 50.00**						
	Time 1	Time 2				
**Genus**	Av.Abund	Av.Abund	Av.Diss	Diss/SD	Contrib%	Cum.%
Merulina	1.95	3.92	3.67	1.3	7.34	7.34
Montipora	5.83	4.01	3.4	1.21	6.79	14.14
Acropora	2.24	0.86	2.92	1.29	5.84	19.98
Pavona	2.09	2.2	2.23	1.24	4.47	24.45
Favites	0.95	1.59	2.05	1.42	4.1	28.55
Diploastrea	0.82	0.89	2.05	0.7	4.1	32.65
Goniastrea	1.76	1.91	1.9	1.33	3.8	36.45
Porites	1.81	2.55	1.9	1.22	3.79	40.24
Pachyseris	1.25	1.91	1.89	1.35	3.79	44.02
Platygyra	0.69	1.5	1.89	1.33	3.78	47.8
Hydnophora	0.48	1.02	1.67	0.99	3.34	51.15
Echinopora	0.28	1.07	1.59	0.98	3.17	54.32
Echinophyllia	0.18	1	1.57	0.91	3.13	57.45
Mycedium	0.96	0.4	1.51	1.06	3.02	60.47
Pectinia	2.85	2.75	1.51	1.19	3.01	63.49
Dipsastraea	0.94	0.75	1.29	1.46	2.58	66.07
Oxypora	0.84	0.12	1.25	0.79	2.5	68.57
Fungia	0.59	0.75	1.25	1.19	2.5	71.06
Galaxea	0.82	0.89	1.18	1.33	2.35	73.42
Symphyllia	0.2	0.74	1.13	0.9	2.26	75.68
Plerogyra	0.73	0.13	1.1	0.97	2.19	77.87
Millepora	0.59	0.02	0.92	0.56	1.84	79.71
Lobophyllia	0.3	0.46	0.88	0.83	1.75	81.47
Podabacia	0.19	0.44	0.79	0.72	1.57	83.04
Pocillopora	0.42	0.28	0.78	0.81	1.57	84.61
Goniopora	0	0.44	0.64	0.63	1.29	85.89
Psammocora	0.33	0.16	0.62	0.49	1.25	87.14
Lithophyllon	0.27	0.21	0.58	0.65	1.15	88.29

Cyphastrea	0.25	0.23	0.56	0.71	1.13	89.42

**Time periods 1 & 3**						
**Average dissimilarity = 55.60**						
	Time 1	Time 3				
**Genus**	Av.Abund	Av.Abund	Av.Diss	Diss/SD	Contrib%	Cum.%
Montipora	5.83	2.76	4.73	1.32	8.51	8.51
Diploastrea	0.82	2.49	3.88	1.04	6.98	15.49
Merulina	1.95	4.02	3.5	1.47	6.3	21.79
Platygyra	0.69	2.72	3.19	1.64	5.74	27.53
Acropora	2.24	0.52	2.98	1.28	5.35	32.88
Goniastrea	1.76	2.7	2.37	1.27	4.27	37.15
Echinophyllia	0.18	1.57	2.19	1.18	3.93	41.08
Favites	0.95	1.99	2.17	1.72	3.91	44.99
Pectinia	2.85	1.86	1.88	1.3	3.39	48.37
Porites	1.81	1.2	1.82	1.25	3.27	51.64
Pavona	2.09	1.78	1.8	1.22	3.23	54.87
Podabacia	0.19	1.19	1.69	1.08	3.04	57.91
Oulophyllia	0	1.04	1.6	0.74	2.89	60.8
Dipsastraea	0.94	1.26	1.59	1.38	2.87	63.66
Galaxea	0.82	1.29	1.46	1.34	2.63	66.3
Pocillopora	0.42	1.13	1.42	1.4	2.55	68.85
Mycedium	0.96	0.3	1.4	0.99	2.53	71.38
Pachyseris	1.25	1.5	1.39	1.38	2.5	73.88
Oxypora	0.84	0	1.18	0.74	2.12	76
Hydnophora	0.48	0.51	1.1	0.77	1.98	77.98
Plerogyra	0.73	0	1.04	0.93	1.88	79.86
Fungia	0.59	0.45	1.04	0.98	1.87	81.73
Psammocora	0.33	0.53	1.02	0.61	1.83	83.56
Echinopora	0.28	0.59	0.97	0.89	1.74	85.3
Cyphastrea	0.25	0.63	0.96	1.14	1.73	87.03
Symphyllia	0.2	0.59	0.9	0.91	1.62	88.65
Millepora	0.59	0	0.89	0.54	1.61	90.26
